# Monetary costs of Alzheimer’s disease in China: protocol for a cluster-randomised observational study

**DOI:** 10.1186/s12883-017-0802-9

**Published:** 2017-01-25

**Authors:** Fangyu Li, Shuoqi Chen, Cuibai Wei, Jianping Jia

**Affiliations:** 1 Department of Neurology, Xuan Wu Hospital, Capital Medical University, Beijing, People’s Republic of China; 2 Center of Alzheimer’s Disease, Beijing Institute for Brain Disorders, Beijing, People’s Republic of China; 3 Beijing Key Laboratory of Geriatric Cognitive Disorders, Beijing, People’s Republic of China; 4 Neurodegenerative Laboratory of Ministry of Education of the People’s Republic of China, Beijing, People’s Republic of China

**Keywords:** Alzheimer’s, Dementia, Cost of illness, Healthcare cost, Observational study

## Abstract

**Background:**

Alzheimer’s disease (AD) is the most common type of dementia. International multilateral cost-of-illness (COI) studies have revealed that the cost of treating this disease is huge, which places a significant burden on patients’ families and their healthcare systems. However, no such studies have been conducted in China. This study estimates the monetary costs of patients with AD in mainland China.

**Methods:**

This study planned to start in October 2015 and to finish in March 2016. It covered 30 provincial, municipal, and autonomous regions in mainland China. The sites and research centres in each region were selected randomly. The participating sites include Tier 3 hospitals, psychiatric hospitals, geriatric hospitals, nursing homes, and residences. More than 2500 patients with AD and their caregivers from all of the 81 research centres will be enrolled to fulfil the calculated sample size. The monetary costs of AD, which include direct medical costs, direct non-medical costs, and indirect costs, are being collected using the electronic medical record system and residence health system at each site; face-to-face interviews are being performed when necessary. Descriptive statistics will be used to summarise the patient characteristics and generalised linear models will be developed to calculate the costs.

**Results:**

The main findings will include national and per patient annual monetary costs of AD in China.

**Conclusions:**

To the best of our knowledge, this is the first large-scale cluster-randomized observational study to estimate the economic burden of AD in Chinese patients. The methodology used was based on China’s current healthcare system and is suitable for the purpose of the study. Because the burden of AD on patients, families, healthcare providers, and society is substantial and increasing, it is important and necessary to understand the economic burden caused by this disease.

**Trial registration:**

Our trial was retrospectively registered on ClinicalTrials.gov, NCT02694445, registered on 02/26/2016

## Background

The Chinese population is rapidly aging. The sixth national population census showed that there were 178 million people aged ≥ 60 years in China, accounting for 13.26% of the total population [1]. The incidence of senile dementia (age of onset ≥65 years) is ~6%, with Alzheimer’s disease (AD) being the most common form, accounting for ~65% of cases [2–5]. In 2014, an epidemiological study that encompassed 30 cities/towns and 45 rural areas from 5 representative Chinese provinces demonstrated that the incidence of dementia and AD in people aged ≥65 was 5.14 and 3.21%, respectively. Furthermore, the prevalence was significantly higher in rural areas compared to urban populations [6].

The monetary costs of AD have been studied worldwide. International multilateral cost-of-illness (COI) studies can identify and measure all of the costs associated with any specific disease including the direct, indirect, and intangible costs; [7] such studies were designed to provide an estimate of the economic burden of disease [7]. A recent study indicated that the cost of AD is substantial and increases with disease severity [8]. Between 2009 and 2010, a cross-sectional survey conducted in the United Kingdom, which included 249 AD patients from 18 cities, revealed that the total monetary costs of AD were ~ £4000 per citizen every 3 months [9]. Moreover, the annual costs associated with AD were $100 billion in the United States, of which $24.6 billion was for healthcare (including caregiver burden and the cost of medical care) [10]. In 2014, a French study showed that the average total monthly costs of AD were €2450 using the proxy good method, and €3102 using the opportunity cost method [11]. In the same year, a study in Finland indicated that the medical costs of an AD patient were €23,059 per person per year [12]. Thus, AD creates a heavy burden to both patients’ families and society and is also a huge challenge to economic development and elderly healthcare. However, to date, studies on the monetary costs of AD have mostly been performed in developed countries such as the United States and United Kingdom, and there have been no large-scale national surveys on the economic burden caused by this disease in developing countries such as China. Because the burden of AD on patients, families, healthcare providers, and society is substantial and increasing [13, 14], our large-scale national study in China is important and necessary.

## Methods

### Study design

**Fig. 1:**
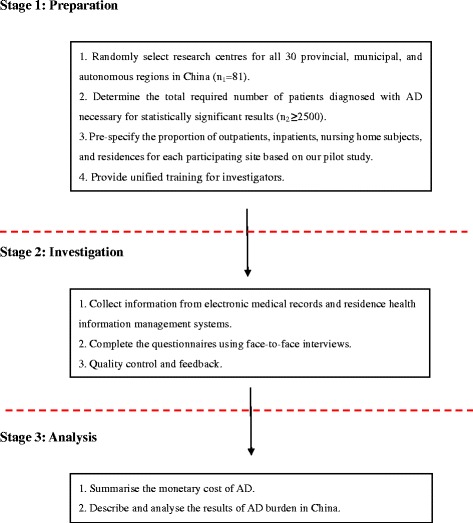
Flow chart of the monetary costs of Alzheimer’s disease in China. *Note:* n_1_, the number of research centres; n_2_, the number of patients with AD

This study is a multicentre study with a large sample size that began in October 2015. Before beginning the study, the protocol and case report form (CRF) were designed using an operator manual, and advice was sought from international scholars. The study design and procedures are illustrated in Fig. 1.

### Distribution of investigation sites and inclusion criteria

This study covered 30 provincial, municipal, and autonomous regions in mainland China, with the exception of Tibet, Hong Kong, and Macao. The target sites included Tier 3 hospitals, mental health centres/psychiatric hospitals, gerontology hospitals, nursing homes/gerocomiums/care facilities, and both urban and rural residences. The study sites in each region were selected randomly. However, each site must meet the following pre-specified criteria: (1) Tier 3 general hospitals, mental health centres, psychiatric hospitals or gerontology hospitals with memory clinics, dementia specialists, psychological evaluation divisions, AD-relevant diagnostic equipment (e.g., computed tomography [CT], 1.5 T or above magnetic resonance imaging [MRI]), and laboratories with the ability to examine samples that may aid in the diagnosis of AD. (2) Nursing homes/gerocomium/care facilities with dementia specialists, >100 beds, and the ability to admit patients with AD. (3) Residences with >500 residents, a residence health system (e.g., electronic health records), active resident or village committees, and staff who can assist with the study. (4) Patients who fully understand the protocol and agree to participate in the study.

**Fig. 2:**
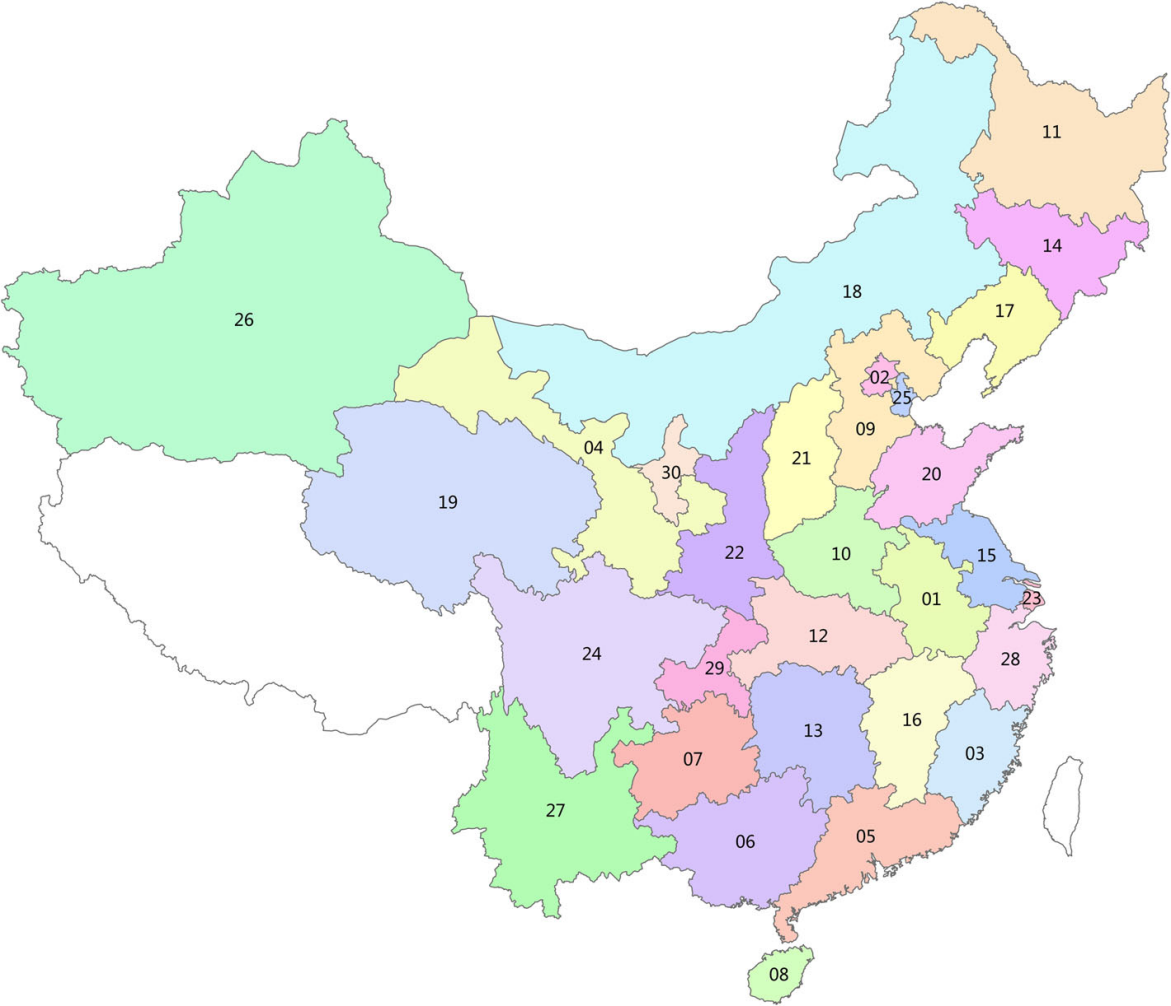
The distribution of investigational centres in China. *Notes:* 01, Anhui Province; 02, Beijing City; 03, Fujian Province; 04, Gansu Province; 05, Guangdong Province; 06, Guangxi Autonomous; 07, Guizhou Province; 08, Hainan Province; 09, Hebei Province; 10, Henan Province; 11, Heilongjiang Province; 12, Hubei Province; 13, Hunan Province; 14, Jilin Province; 15, Jiangsu Province; 16, Jiangxi Province; 17, Liaoning Province; 18, Inner Mongolia Autonomous; 19, Qinghai Province; 20 Shandong Province; 21, Shanxi Province; 22, Shaanxi Province; 23, Shanghai City; 24, Sichuan Province; 25, Tianjin City; 26, Xinjiang Autonomous; 27, Yunnan Province; 28, Zhejiang Province; 29, Chongqing City; 30, Ningxia Autonomous

The study included 81 research centres that meet the inclusion criteria (Fig. 2).

### Criteria for AD patients

Because we investigate the direct and indirect monetary costs of AD, strict inclusion and exclusion criteria for AD are essential. The following patients are being included: those who are newly diagnosed with AD in this study or who have been previously diagnosed, patients aged ≥ 60 years with normal or corrected vision and hearing, and those who are able to provide an informed consent form signed by himself/herself or a legal guardian. Dementia is diagnosed according to the criteria described by the Diagnostic and Statistical Manual of Mental Disorders, Fourth Edition, Text Revision (DSM-IV-TR) [15]. The diagnosis of AD is made using the National Institute of Neurologic and Communicative Disorders and Stroke and the Alzheimer’s Disease and Related Disorders Association (NINCDS-ADRDA) [16]. The diagnosis also required to meet the following: a Modified Hachinski Ischemic Scale (MHIS) score ≤ 4 [17], an Activity of Daily Living Scale (ADL) score ≥ 23 [18], and a Geriatric Depression Scale (GDS) score < 11 [19]. Neuroimaging MRI is used to support the AD diagnosis (atrophy in the medial temporal lobe and a white matter lesion score ≤ 2 according to the Fazekas criteria) [20, 21]. The severity of AD is estimated according to the mini-mental state examination (MMSE) scale [22] (for illiterate dementia mild, 21–24 points; moderate, 11–20; severe, ≤10. For literate dementia: mild, 16–19 points; moderate, 8–15; severe, ≤7). The diagnosis of AD is confirmed by brain CT or MRI scans and laboratory tests to rule out any significant comorbidities. Patients will be excluded if they meet any of the following criteria: presence of vascular dementia or dementia caused by other factors such as major depression or other major psychiatric illnesses, thyroid dysfunction, encephalitis, multiple sclerosis and other dementias such as frontotemporal dementia (FTD) [23], dementia with Lewy bodies (DLB) [24], and Parkinson’s dementia (PD); [25] when MRI and laboratory tests (e.g., blood cell counts, liver and renal function tests) did not support or rule out a diagnosis of AD; and a history of alcoholism or drug abuse.

### Sample size

The sample size was calculated using the equation, $n=\frac{U_{1-\alpha /2}^2{S}^2}{\delta^2}$ where *S* was the stand division and *δ* was the allowable error. The planned sample size was generated based on previous data obtained from an economic evaluation of an AD-type dementia study [26]. Based on the standard deviation (SD) of AD-related costs per patient per year (11,037) and an allowable error of 500, the total sample size required was 1,872. Therefore, the planned total sample size in this study was ~2,500 to allow for a defective rate of 25%. Before starting the study, information was surveyed from all randomly selected participating sites, including the number of dementia physicians at each site, the number of AD patients, and the proportion of outpatients and inpatients in the last year. Based on these data 20–40 patients with AD will be assigned for each site. The sites include hospitals (Tier 3 hospitals, gerontology hospitals, mental health centres and psychiatric hospitals), nursing homes, gerocomiums, care facilities, and residences.

#### Cost classification

The AD-associated costs are defined as direct medical costs, direct non-medical costs, and indirect costs (Table 1).

**Table 1:** Classification of AD-associated costs

AD-associated costs	Subgroup costs
Direct medical cost	outpatient
	hospitalisation
	out-of-pocket
Direct non-medical cost	transportation, accommodation, and meals
	nourishment
	healthcare equipment
	formal care
Indirect cost	informal care
	intangible cost

#### Direct medical costs

The direct medical costs include outpatient costs, hospitalisation costs, and out-of-pocket expenses for healthcare medications. Outpatient costs include the reason for the visit (due to AD, AD-related symptoms, or comorbidities), the number of outpatient visits in the past 3 months, and the cost of the last clinic visit in the same 3-months period. The cost of clinic visits includes registration (general physicians or specialists), diagnostic procedures (e.g., consultation, scale evaluation), imaging (e.g., CT, MRI, SPECT, and PET), laboratory tests, medication, and non-drug treatment (such as rehabilitation treatment). The outpatient medication costs are divided into three categories: anti-dementia drugs, medications for the treatment of AD-related symptoms (e.g., sleep disorders, psychiatric symptoms, and gastrointestinal symptoms caused by medications), and the treatment of comorbidities. For anti-dementia drugs the specific medications used and their corresponding costs are also investigated. For comorbidity-associated costs, the types of comorbidity and the associated costs are recorded. Investigations into the cost of hospitalisation included the reason for hospitalisation (AD, AD-related symptoms, or comorbidities), how many times a patient was hospitalised in the past 12 months, and expenditures on the last hospitalisation within the past 12 months. The hospitalisation expenditure includes the cost of the ward bed, formal care, examinations, laboratory tests, treatment (such as rehabilitation, massage, and others), and medications. The term “self-bought medications” means that the patient or their caregivers purchased the medications outside the hospital (out-of-pocket spending). Questions include how many times medications were purchased in the past month and the total expenditure during the same period. The above data also include information regarding the patients’ payment method (including medical insurance for urban workers or town residents, rural cooperative medical insurance, commercial insurance and self-funded) and any out-of-pocket expenses.

#### Direct non-medical costs

The direct non-medical costs include the cost of transportation, accommodation, and meals when visiting a physician, the cost of nourishment and healthcare equipment in the patient’s daily life, and formal care fees. The cost of daily nourishment, such as calcium, vitamin, protein powder, and fish oil supplements, include the number of times items were purchased and the amount spent in the last 12 months. The cost of healthcare equipment refers to supplies, such as positioning watches, bracelets, and wheelchairs, used in the last 12 months. Formal care costs are defined as the cost of nursing care given by professional caregivers for patients in nursing homes/gerocomiums/care facilities or at home, including the number of hours of care and the average monthly expenses [27].

#### Indirect costs

Indirect costs include monetary loss caused by the patients’ inability to work, reduction of informal caregivers’ income, treatment of the mental suffering of caregivers, and treatment of unexpected injuries in AD patients or their caregivers. Because the AD patients in this study are aged ≥ 60 years, most of whom are retired as per China’s national regulations, their major source of income is retirement pensions. Therefore, the decrease in a patients’ income is not considered, because it is independent of patients’ health status. Informal caregivers are mostly the relatives or unpaid nonrelatives of patients with no agency affiliation [28]. Investigation of the informal caregivers include the length of time (hours) provided by informal caregivers; the relationship between the caregivers and AD patients; sex, age, and monthly job losses for employed subjects (hours); and monthly salary. Cost for the treatment of mental suffering of caregivers refers to any symptoms triggered or worsened by taking care of AD patients over the past 12 months. The monetary loss caused by injuries to AD patients includes outpatient, examination, medication, transportation, and accommodation costs.

#### Others

To understand the relationship between AD costs and other factors, parameters such as social demographics and disease details are also investigated. The social demographics include sex, age, education level, marital status, occupation, salary, and the number of children. The disease details include age at onset of AD, clinical evaluations (based on ADL, GDS, MMSE and neuropsychiatric inventory [NPI] [29]), and comorbidities (e.g., stroke, diabetes, heart disease, hypertension, hyperlipidemia, lung diseases, cancer, arthritis, and other diseases of the nervous system).

### Data collection procedure

Electronic medical record systems are being used to identify elderly patients diagnosed with AD among outpatients (in the last 3 months), inpatients (in the last 12 months), those living in urban residences and rural residences with their families, and residents of nursing homes, gerocomiums, or care facilities. The diagnosis of AD is re-confirmed by dementia specialists before the start of the study. The intensive dementia training program conducted in national and regional centres included both theory and practice. It comprised the study methods, assessment of the scales and internationally accepted diagnostic criteria of dementia and its subtypes, neuroimaging (CT and MRI), and standard procedures for diagnosis. Investigators are contacting patients and re-confirm the accuracy of the AD diagnosis according to the inclusion and exclusion criteria. Neuroimaging and laboratory tests will be performed when necessary. New patients are also being enrolled during the study period. Face-to-face questionnaires will be administered to AD patients and their caregivers when the data and information could not be collected through electronic medical record or residence health systems. The surveys are being administered by investigators to ensure the accuracy of the diagnosis and data.

### Measures

#### Total cost per patient per year

The total cost per patient per year is determined by adding the annual direct medical costs, direct non-medical costs, and indirect costs for each patient. When calculating the cost of the recuperation fee for patients who required nursing care in nursing homes, gerocomiums, or care facilities, 8% of the basic cost is subtracted to reflect the AD patient’s basic cost of living [30]. Two approaches are being used to estimate the monetary loss of informal caregivers. For the informal caregivers who have no stable job before taking care of the AD patients, the average labour market income reported by each research centre is being multiplied by the amount of time spent being a caregiver to obtain the monetary loss. For individuals with a stable job before taking care of the AD patient, the monetary loss is being obtained by multiplying the hourly salary before they stop working by the time spent (hours) being a caregiver. All of the costs will be converted into the average annual cost.

#### Current total number of patients with AD in China

A model is established to estimate the total number of Chinese patients with AD in 2015 based on the literature. The total number of patients with AD is defined as M and the percentage of AD patients who received anti-dementia treatment is defined as d%. Therefore, the number of anti-dementia treated patients (M_1_) is M × d%, and the number of patients not treated for dementia (M_2_) is M × (1–d)%. Based on previous reports, the percentage of AD patients who are treated for dementia is 10–30% [31, 32]. Epidemiological studies have revealed that the number of AD patients in China aged ≥ 60 years in 1990, 2000, and 2010 was 1.93 million, 3.71 million, and 5.69 million, respectively [8, 33, 34]. A linear regression model is used to estimate the age-specific prevalence of AD in 2015 by recording the prevalence of AD among different-aged patients. By multiplying the percentage of AD among elderly individuals aged ≥ 60 years by the total national elderly population (aged ≥ 60 years) in 2015, we obtain the estimated total number of AD patients of China, which is defined as M.

#### Per capita annual cost of AD patients (who accepted or did not accept treatment)

The cost associated with treating dementia patients (F_1_) is divided into two groups: “outpatient costs” and “hospitalised or inpatient costs.” F_1_1_ is defined as the average annual cost in the inpatient group (average annual cost × the number of inpatients annually), and F_1_2_ is the average annual cost in the outpatient group (average annual cost × the number of outpatients annually). Patients described as not accepting treatment are those who did not select either “outpatient service costs” or “hospitalised costs” in their questionnaire. F_2_ is defined as the average annual cost per AD patient who did not accept treatment.

#### Nationwide cost due to AD

A pilot study is performed, which reveal that the average annual ratio of inpatients to outpatients in different hospitals is ~1:6. Hence, the number of patients who undergo treatment nationwide (M_1_) is multiplied by 1/7 or 6/7 to obtain the number of hospitalised patients with AD (M_1_1_) and the number of outpatients with AD (M_1_2_), respectively. Thus, the total national annual cost of patients who accept treatment is M_1_1_ × F_1_1_ + M_1_2_ × F_1_2_. M_2_ is defined as the number of patients who did not accept treatment, and this value is multiplied by F_2_ to obtain the total annual national cost of patients who do not accept treatment. Therefore, the total annual national cost of patients with AD is M_1_1_ x F_1_1_ + M_1_2_ x F_1_2_ + M_2_ x F_2_. To estimate the costs attributable to AD, generalised linear models (GLMs) related to coexisting conditions (stroke, diabetes, heart disease, hypertension, lung disease, cancer, arthritis, and psychiatric problems) and demographics (sex, age, educational level, marital status, and household income) are used. Moreover, we estimate the total numbers of people who will be living with AD in 2020, 2030, 2040, and 2050 to predict the total annual cost of AD in future decades. Few large-scale national surveys of the monetary cost or economic burden of AD have been conducted in China, or indeed in Asia. To remedy this lack of data, we also systematically review COI studies of AD that report annual costs per patient. This will enable estimation of the proportion of the global burden of AD costs faced by China. Furthermore, it will enable comparison of the economic burden of AD in developed and developing countries.

### Statistical analysis

#### Descriptive statistics

Descriptive statistics (means ± SDs or frequencies) will be used to summarise the patient characteristics, including age, sex, years of formal education, time since AD diagnosis, marital status, living location (urban or rural), number of children, baseline MMSE score, baseline ADL score, number of comorbidities, number of caregivers, and information about the caregivers (such as sex, age, relationship to patient, and employment status). Mean values and ranges will be also presented because cost data are commonly positively skewed and the median cost could be zero for highly skewed data.

#### Calculation of costs due to AD

Different distributions will be considered for the monetary costs associated with AD, including normal, lognormal, exponential, and gamma. To compare the costs of different subgroups, *t*-tests or Kruskal-Wallis analysis of variance will be used. For base case analyses, GLMs will be developed using the gamma distribution with a log-link function [35]. Models will be developed with the total cost per person per year as the dependent variable, and independent variables will be selected using a backward selection method (*P* > 0.05 for removal), with patient age, patient sex, and MMSE severity all forced to remain in the model. Considering the diverse of research centres sites, we also added the sites as a covariant in the analysis. The variables used for backward selection will be years of formal education, time since AD diagnosis, marital status, location of residence (urban or rural), number of children, baseline MMSE score, baseline ADL score, the number of caregivers, the number of comorbidities, and caregiver demographics.

#### Sensitivity analyses

Sensitivity analyses will be conducted to examine the robustness of the results from the base case model and explore correlations between certain factors that could impact each other. First, the total costs of dementia will be estimated using the prevalence rates derived from a systematic review. Then the minimum and maximum salaries (rather than the average) will be used to estimate the relative costs. The total costs will be estimated by assuming that 70 and 99% of patients with AD are living at home, instead of 86% in the primary option. Furthermore, different costing caregiver time will be used to estimate the total cost. All of the analyses will be performed using R software version 3.2.3.

### Quality control

The questionnaire and standard operating standards procedures were formulated based on previous studies and by consulting relevant experts internationally and domestically. All of the staff working on this study were trained to ensure that standard operating procedures were followed, and all of them were required to pass an examination before beginning data collection. A pilot study was conducted in five randomly selected hospitals and residences to evaluate the feasibility and reliability of the questionnaire and to identify any potential problems.

## Results

This study began in October 2015 and will finish in March 2016. The main findings will be the per patient and national annual monetary costs of AD in China. The costs include direct medical, direct non-medical, and indirect costs. Data of the subgroup costs will also be reported. Social demographics, illness and treatment of AD, scale scores, and comorbidities will be shown in a table at last.

## Discussion

To the best of our knowledge, this is the first study on AD cost that encompassed nearly all of the provincial, municipal, and autonomous regions in mainland China. To complete this survey, it is crucial to prepare a thoughtful and well-designed protocol. In the study design phase, we referred to an international popular survey method and adjusted it to China’s national conditions. We also tested the protocol in a pilot study; the results suggested that this protocol can be used in China.

This protocol includes measures specific to problems that exist in China. First, China has the largest population of any country, and the varying economic development among regions [36] might result in introduction of bias. To avoid this, the study includes 30 of the 33 provincial, municipal, and autonomous regions in mainland China. This ensures that both poor and rich areas are covered to balance the gap between them because people in poor areas may have more health problems [37]. Second, in China, hospitals with physicians for patients with AD include Tier 3 general hospitals, gerontology hospitals, mental health centres, psychiatric hospitals, and other institutions such as nursing homes, gerocomiums, and care facilities [36]. Therefore, all types of institutions are included in the current investigation to ensure representation of the diversity of AD costs among hospitals and institutions. The hospitals and institutions are selected randomly to reduce the test error. Third, the target population of this survey is patients with AD. Accurate diagnosis of AD is required for calculation of the true monetary cost of the condition. AD patients diagnosed according to the NINCDS-ADRDA criteria are enrolled in the study. In particular, to ensure accuracy, the diagnosis of patients diagnosed with AD prior to the start of this study was re-confirmed. In addition, the project team comprises a group of dementia physicians, who are trained in the diagnostic processes for dementia and AD to guarantee the quality and consistency of the diagnosis. Fourth, for accurate determination of the costs associated with AD patients, electronic records systems are used to determine the costs of all treatment items. This measure guarantees the absence of error. Therefore, the current protocol facilitates determination of the cost burden of AD patients in China.

We carefully consider cost classifications for the total cost of AD. Cost in the present study includes direct medical costs, direct non-medical costs, and indirect costs. The cost classification used was in accordance with those that are commonly used internationally [11, 38]. This strategy is intended not only to ensure that the results cover all aspects of costs for AD patients but also to enable comparison with findings from developed countries. In addition, the cost of AD includes both AD patients and their caregivers. Our previous study revealed that 84.9% of AD patients are cared for by non-professional caregivers at home [39]. This is because China faces a shortage of nursing homes, and the Chinese tradition of filial piety mandates that when they lose the ability to live independently, patients should be cared for by their families. Therefore, it is important to include caregivers in calculating AD costs. These procedures are conducted to ensure the accuracy, reliability, and credibility of data on AD in China.

## Conclusion

In conclusion, this study provides an important basis for calculation of the annual cost of AD, will assist the understanding of the government and policy makers of the position and role of healthcare in the national economy and social development, and will enable formulation of a national plan for dementia. In addition, the results will facilitate evaluation of the effectiveness of the national funds allocated to AD research.

## Abbreviations

AD: Alzheimer’s disease
ADL: Activity of daily living scale
COI: Cost of illness
CRF: Case report form
CT: Computed tomography
DLB: Dementia with Lewy bodies
DSM-IV-TR: Diagnostic and Statistical Manual of Mental Disorders, Fourth Edition, Text Revision
FTD: Frontotemporal dementia
GDS: Geriatric depression scale
GLMs: Generalised linear models
MHIS: Modified Hachinski Ischemic Scale
MMSE: Mini-mental state examination
MRI: Magnetic resonance imaging
NINCDS-ADRDA: National Institute of Neurologic and Communicative Disorders and Stroke and the Alzheimer’s Disease and Related Disorders Association
NPI: Neuropsychiatric inventory
PD: Parkinson’s dementia
SD: Standard deviation
